# A Rare Case of Neuromyelitis Optica Spectrum Disorder in Patient with Sjogren's Syndrome

**DOI:** 10.1155/2014/158165

**Published:** 2014-11-19

**Authors:** Supat Thongpooswan, Bikash Chapagain, Sabiha Bandagi

**Affiliations:** Icahn School of Medicine at Mount Sinai, Queens Hospital Center, Jamaica, NY 11432, USA

## Abstract

We report a 48-year-old female with the history of Sjogren's syndrome who presented with 3-week history of tingling, numbness, and shooting back, waist, and bilateral leg pain and numbness in the pelvic region with urinary and bowel incontinence. Physical examination was remarkable for reduced motor power in both lower extremities with spasticity. Sensory deficit was noted at the T6 level. Laboratory investigation revealed elevated ESR and CRP and positive serum antiaquaporin-4 IgG. Thoracic and lumbar magnetic resonance imaging revealed abnormal patchy areas, leptomeningeal enhancement through the thoracic cord extending from T3 through T6 levels, without evidence of cord compression. Impression of neuromyelitis optica spectrum disorder was made and patient was treated with methylprednisolone intravenously followed by tapering oral prednisone. Neurological symptoms gradually improved with resolution of bowel and urinary incontinence. In a patient with Sjogren's syndrome who presents with neurological complaints, the possibility of neuromyelitis optica or neuromyelitis optica spectrum disorder should be considered. Awareness of the possibility of CNS disease is important due to the serious nature of CNS complications, some of which are treatable with immunosuppressants. Our patient with Sjogren's syndrome who presented with myelopathy benefited from early recognition and institution of appropriate therapy.

## 1. Introduction

Sjogren's syndrome (SS) is a chronic autoimmune disease characterized by lymphocytic infiltration of exocrine glands. SS coexists with other rheumatic conditions such as rheumatoid arthritis or Systemic lupus erythematosus (SLE). Neurological involvement in SS can involve both the central and peripheral nervous systems. Symptomatic Central nervous system (CNS) involvement can be in the form of myelopathy, optic neuritis, seizures, and cognitive dysfunction. Distal paresthesias, cranial neuropathy, and mononeuritis multiplex suggest peripheral nervous system involvement. We report a rare case of SS complicated by neuromyelitis optica spectrum disorder (NMSD).

## 2. Case Presentation

This is a case of 48-year-old Guyanese female who presented with 3-week history of tingling, numbness, and shooting pain in the back, waist, and both legs and numbness in the pelvic region. Patient also reported having urinary and bowel incontinence which started 3 days prior to presentation.

Seven years back patient initially presented with inflammatory arthritis of both wrists and interstitial lung disease and few years later developed sicca symptoms with positive SSA/Ro antibody. Lip biopsy however was reported as negative for SS. Patient was being treated with azathioprine 100 mg daily and prednisone 10 mg oral daily.

At presentation, vital signs were normal. Ophthalmologic exam revealed normal pupils and visual acuity and extraocular muscles were intact. Neurological examination revealed normal cranial nerves and upper extremities strength and sensation, and there was no nuchal rigidity. Bilateral lower extremities revealed increased spasticity and motor power of 4/5 in quadriceps and hamstrings. Sensory deficit was noted at the level of T6. Deep tendon reflexes were preserved except for the plantar reflexes. Patient refused rectal examination.

Laboratory investigation revealed hemoglobin 14.2 g/dL, white blood cell count of 4.9 K/mcl, and platelet 239 K/mcl. Renal panel and liver function test were within normal limits. C3 and C4 were normal. ESR 42 mm/h, CRP 1.07 mg/dL, antinuclear antibody, anti-double-stranded DNA, anticyclic citrullinated peptide, rheumatoid factor, anticardiolipin IgM, IgG, lupus anticoagulant, syphilis IgG, HIV antibody, and human T-lymphotropic virus (HTLV) I/II were negative. Serum antiaquaporin-4 IgG was positive.

Thoracic and lumbar magnetic resonance imaging (MRI) revealed abnormal patchy areas, leptomeningeal enhancement through the thoracic cord extending from superior T3 through inferior T6 levels, with no evidence of cord compression. Brain MRI revealed no evidence of multiple sclerosis or optic neuritis.

Cerebrospinal fluid analysis showed WBCs of 19/cu mm (2% of polynuclear cell and 83% of lymphocyte), RBCs 600, protein 54 mg/dL, and glucose 89 mg/dL. VDRL test was negative.

Diagnosis of NMSD was made. Patient was treated with methylprednisolone intravenously 1 gram daily for 5 days followed by tapering oral prednisone. Azathioprine was increased to 150 mg daily. Patient's neurological symptoms gradually improved with resolution of bowel and urinary incontinence.

## 3. Discussion

SS is an autoimmune disease in which there is mononuclear infiltration of exocrine gland and salivary and lacrimal glands resulting in xerophthalmia and xerostomia, also known as sicca syndrome. Blood tests are usually positive for SSA/Ro or SSB/La antibodies. Rose Bengal test, Schirmer test, and saliva gland/lip biopsy can be done to support diagnosis.

Neurological involvement could be observed in approximately 20–25% of cases of SS. Neurological manifestation of SS involves central and peripheral nervous system. Involvement can range from focal or multifocal central lesions to dementia and conditions that mimic MS such as neuromyelitis optica (NMO) or NMSD. Spinal cord involvement is an infrequent yet potentially devastating manifestation of systemic autoimmune disease [1].

NMO is characterized by two absolute criteria; (1) optic neuritis and (2) acute myelitis plus 2 of the 3 following supportive criteria: (1) brain MRI not meeting criteria of MS, (2) spinal cord MRI extending over three or more vertebral segments, and (3) NMO-IgG positive. However, transverse myelitis and positive NMO-IgG without optic neuritis like in our case, which does not fully meet the NMO criteria, have been categorized as NMSD [2].

NMO or NMSD have been associated with rheumatic conditions mainly SLE and SS. There have been reports of SS complicated by NMSD including long extended spinal cord lesion (LESCL). Min et al. and Estiasari et al. reported that the rates of central nervous system involvement in SS patients were with LESCL positive for the anti-AQP4 antibody in East Asia 42% and 18%, respectively [3, 4]. However, SS complicated by NMO seems to be rare in non-East Asian regions. Kolfenbach et al. reviewed English literatures and found 21 cases of overlap SS and NMO with additional 5 more cases of their own [5]. The etiology of this association between autoimmune disease and NMO is still unclear. However, there is a retrospective blinded serological survey to support the evidence of coexisting NMO in NMSD with positive NMO-IgG occurring with SS rather than as a complication of SS [6].

The first line treatment of acute attack of NMO or NMSD is high dose intravenous methylprednisolone 1000 mg daily for at least three to five days [7]. Plasmapheresis or cyclophosphamide may be considered if there is no clinical improvement with steroid therapy alone [8–10]. Azathioprine, rituximab, mycophenolate mofetil, methotrexate, prednisone, or mitoxantrone can be used as maintenance therapy [7]. However, in this case, the patient was affected with NMSD during administration of azathioprine and prednisone. It is necessary to change to other alternative therapies: rituximab, mycophenolate mofetil, methotrexate, or mitoxantrone [7].

Our case illustrates the association between SS and NMSD. In patient with SS who presents with neurological complaints, the possibility of NMO or NMSD should always be considered because it is a rarely reported occurrence. Awareness of the possibility of CNS disease is important because of the serious nature of CNS complications and because of the fact that some are treatable with immunosuppressive medications. Our patient with SS who presented with myelopathy benefitted from early recognition and institution of appropriate therapy.

## 4. Conclusion

This is a case of NMSD associated with connective tissue disease. The case supports the association between NMSD and SS. Early diagnosis and treatment are critical in decreasing the possibility of irreversible severe neurological dysfunction.
